# A neurocomputational view of the effects of Parkinson’s disease on speech production

**DOI:** 10.3389/fnhum.2024.1383714

**Published:** 2024-05-15

**Authors:** Jordan L. Manes, Latané Bullock, Andrew M. Meier, Robert S. Turner, R. Mark Richardson, Frank H. Guenther

**Affiliations:** ^1^Department of Speech, Language, and Hearing Sciences, Boston University, Boston, MA, United States; ^2^Department of Communicative Disorders and Sciences, University at Buffalo, Buffalo, NY, United States; ^3^Program in Speech and Hearing Bioscience and Technology, Division of Medical Sciences, Harvard Medical School, Boston, MA, United States; ^4^Department of Neurosurgery, Massachusetts General Hospital, Boston, MA, United States; ^5^Harvard Medical School, Boston, MA, United States; ^6^Department of Neurobiology, University of Pittsburgh, Pittsburgh, PA, United States; ^7^Aligning Science Across Parkinson’s (ASAP) Collaborative Research Network, Chevy Chase, MD, United States; ^8^Department of Biomedical Engineering, Boston University, Boston, MA, United States; ^9^Picower Institute for Learning and Memory, Massachusetts Institute of Technology, Cambridge, MA, United States

**Keywords:** Parkinson’s disease, speech, modeling, dysarthria, basal ganglia, deep brain stimulation

## Abstract

The purpose of this article is to review the scientific literature concerning speech in Parkinson’s disease (PD) with reference to the DIVA/GODIVA neurocomputational modeling framework. Within this theoretical view, the basal ganglia (BG) contribute to several different aspects of speech motor learning and execution. First, the BG are posited to play a role in the initiation and scaling of speech movements. Within the DIVA/GODIVA framework, initiation and scaling are carried out by initiation map nodes in the supplementary motor area acting in concert with the BG. Reduced support of the initiation map from the BG in PD would result in reduced movement intensity as well as susceptibility to early termination of movement. A second proposed role concerns the learning of common speech sequences, such as phoneme sequences comprising words; this view receives support from the animal literature as well as studies identifying speech sequence learning deficits in PD. Third, the BG may play a role in the temporary buffering and sequencing of longer speech utterances such as phrases during conversational speech. Although the literature does not support a critical role for the BG in representing sequence order (since incorrectly ordered speech is not characteristic of PD), the BG are posited to contribute to the scaling of individual movements in the sequence, including increasing movement intensity for emphatic stress on key words. Therapeutic interventions for PD have inconsistent effects on speech. In contrast to dopaminergic treatments, which typically either leave speech unchanged or lead to minor improvements, deep brain stimulation (DBS) can degrade speech in some cases and improve it in others. However, cases of degradation may be due to unintended stimulation of efferent motor projections to the speech articulators. Findings of spared speech after bilateral pallidotomy appear to indicate that any role played by the BG in adult speech must be supplementary rather than mandatory, with the sequential order of well-learned sequences apparently represented elsewhere (e.g., in cortico-cortical projections).

## 1 Introduction

The aim of this paper is to provide a neurocomputational view of the planning and execution of speech in Parkinson’s disease (PD) by reviewing the scientific literature within the theoretical framework of two well-developed computational models of speech production: the DIVA and GODIVA models (see Guenther, 2016 for a detailed treatment). PD is the second most common neurodegenerative disorder in the world (Feigin et al., 2020), affecting approximately 8.5 million adults worldwide (World Health Organization, 2023). As the disease progresses, markers of neuropathology (e.g., Lewy bodies) are observed in different areas of the nervous system, proceeding through characteristic *Braak stages* (Braak et al., 2004). This process begins in the lower brainstem and olfactory system (Stage 1); followed by the raphe nucleus and locus coeruleus (Stage 2); the substantia nigra pars compacta (SNc) and basal nucleus of Mynert (Stage 3); the thalamus, amygdala, mesocortex, and allocortex (Stage 4); and finally, neocortex (Stages 5–6). Clinically, PD is characterized by a set of classic motor signs, including bradykinesia, tremor, rigidity, postural instability, and gait disturbances. Speech and voice symptoms are highly prevalent in people with PD (Sapir et al., 2008). Perceptually, the characteristics of speech in PD include monopitch, monoloudness, reduced stress, imprecise consonants, inappropriate silences, short rushes of speech, harsh voice, breathy voice, low pitch, and variable rate (Duffy, 2019), with voice changes typically presenting as the earliest and most prevalent speech symptoms (Logemann et al., 1978; Ho et al., 1998; Harel et al., 2004; Rusz et al., 2011). The constellation of motor speech symptoms of PD reflects a complex interplay of sensory, motor, cognitive, and affective mechanisms (Sapir, 2014).

**Figure 1:**
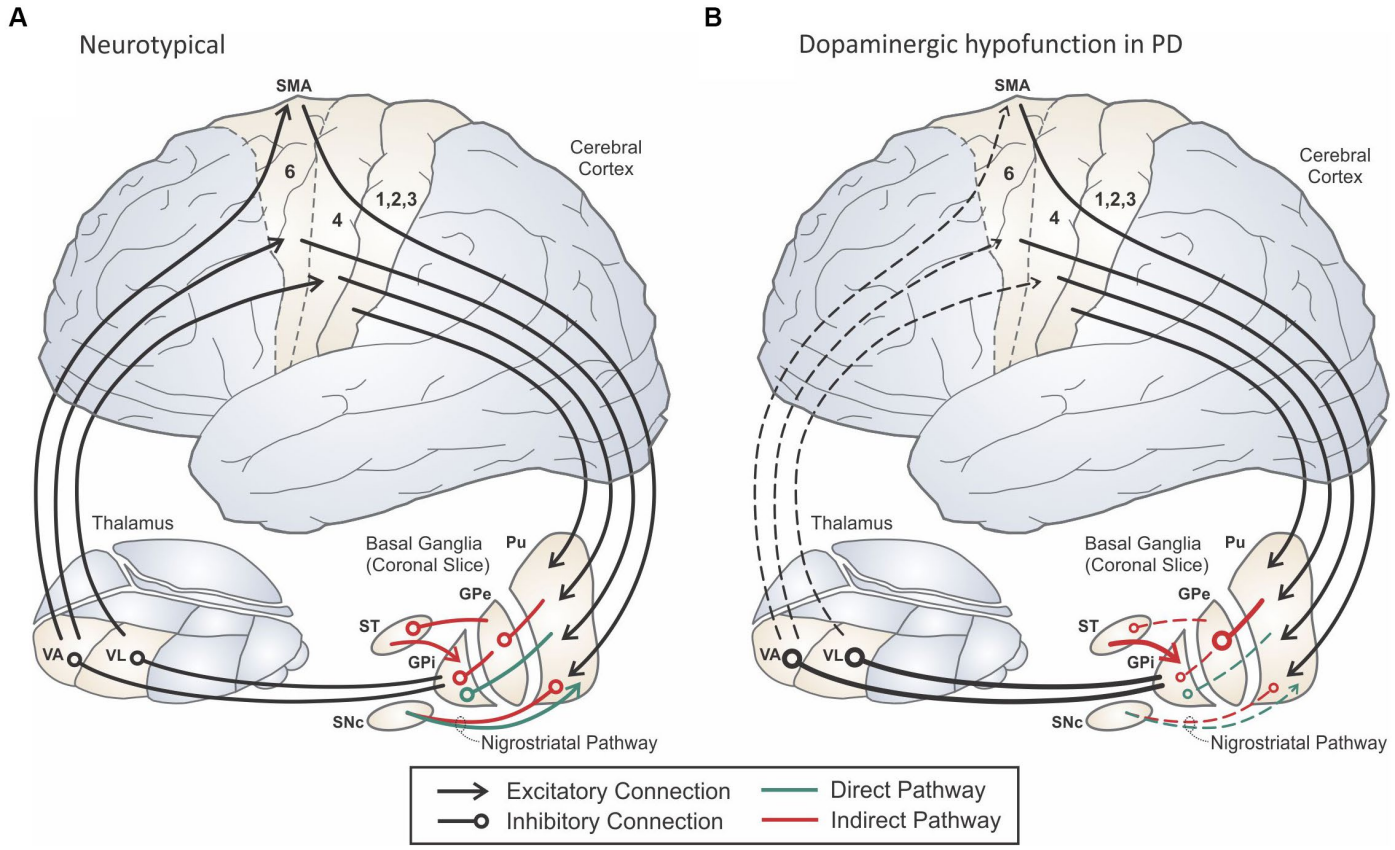
Schematic of the cortico-basal ganglia-thalamo-cortical motor loop in a neurologically normal adult **(A)** and an adult with Parkinson’s disease **(B)**. Adapted from Guenther (2016).

The most well-characterized and devastating effects on motor control in PD arise from pathophysiological changes in the basal ganglia (BG). The BG are functionally organized into sensorimotor, oculomotor, associative, and limbic loops based on their projections to relevant regions of the cerebral cortex (Alexander et al., 1990). Figure 1 shows the classic firing-rate based model of the cortico-BG-thalamo-cortical (hereafter *cortico-BG*) motor loop in a healthy individual (panel A) and in a person with PD (panel B). In PD, the presence of Lewy body pathology and destruction of cells within the substantia nigra pars compacta (*SNc*) occurring during Braak Stage 3 result in the loss of dopaminergic input to the putamen and caudate nucleus of the striatum. These striatal nuclei form the main input structures of the basal ganglia, receiving projections from a wide range of sensory, motor, and cognitive regions of the cerebral cortex. Without sufficient dopaminergic input, cortico-BG loops become dysregulated, leading to changes in the firing rates and firing patterns of neurons within these pathways.

Dopamine has an excitatory effect on striatal neurons in the *direct pathway* (indicated in green in Figure 1) that send inhibitory projections to the internal segment of the globus pallidus (*GPi*) and/or the substantia nigra pars reticulata (*SNr*), which together form the output portion of the basal ganglia. In contrast, dopamine has an inhibitory effect on striatal neurons in the *indirect pathway* (indicated in red in Figure 1). Striatal neurons in the indirect pathway send inhibitory projections to the external segment of the globus pallidus (*GPe*), which in turn inhibits GPi, both directly as well as through inhibiting the subthalamic nucleus (*STN*). GPi neurons project through inhibitory pathways to the thalamus, in particular the ventral anterior (*VA*) and ventral lateral (*VL*) thalamic nuclei, which send excitatory projections to the motor and premotor cortical areas, respectively. The direct pathway is often characterized as *pro-kinetic* since activating a striatal neuron in the direct pathway is thought to have a net excitatory effect on motor cortex: the striatal neuron inhibits GPi, reducing inhibitory GPi output to thalamus and increasing excitatory input to cortex via thalamus. Because the indirect pathway includes an additional inhibitory stage, activating a striatal neuron in the indirect pathway is thought to have a net inhibitory (*anti-kinetic*) effect on motor cortex. A third pathway (not shown in Figure 1) from cortex to STN called the *hyperdirect* pathway has been hypothesized to inhibit large areas of cortex representing possible motor programs before the correct motor program is activated via the direct pathway (Mink, 1996; Nambu et al., 2002; see Turner and Desmurget, 2010 for an alternative view).

Early basal ganglia models of PD focused on the impacts of basal ganglia dysregulation on cortical firing rates (e.g., Albin et al., 1989; Delong, 1990). Within the classic rate model of PD, deficient dopaminergic input results in overactivation of the anti-kinetic indirect pathway and underactivation of the pro-kinetic direct pathway, leading to a net decrease of excitatory input to cerebral cortex from the thalamus (Figure 1B). More recent models have focused on changes to neuronal firing *patterns* and the synchronization of firing rates across neuronal populations (e.g., Levy et al., 2000; Brown, 2003; Magnusson and Leventhal, 2021). These pattern-based models instead describe a pathological increase in synchronized firing between neighboring neurons in the basal ganglia nuclei and their output structures, which interferes with the reliable transmission of signals between thalamus and cortex.

It is well-established that speech production heavily involves the cortico-BG motor loop. Functional neuroimaging studies of speech and vocal tract movements commonly report activity in the putamen (Brown et al., 2009; Chang et al., 2009; Parkinson et al., 2012; Simonyan et al., 2013). Moreover, lesions of the putamen and caudate result in marked changes in speech production and organization (Pickett et al., 1998; Gronholm et al., 2016). STN and GPi are also functionally connected to multiple cortical and subcortical regions within the speech network (Manes et al., 2014).

Intracranial recordings from humans undergoing deep brain stimulation (DBS) lead placement surgery have provided valuable evidence for STN involvement in speech production. Watson and Montgomery (2006) were the first to report basal ganglia recordings during speech production. In line with firing rate models of the basal ganglia, the authors reported drastic *reduction* in firing rate in the STN for the duration of an utterance during a sentence repetition task. However, more recent studies have painted a far more complex picture of STN and GPi function during speech. Neurons both increase and decrease firing rate in the STN in response to visual task-related cues and to speech production (Lipski et al., 2018; Tankus and Fried, 2019; Tankus et al., 2021; Johari et al., 2023), though a robust finding across reports and tasks is that most neurons *increase* firing rate at speech onset in STN (Johari et al., 2023). Studies of non-human primates (*NHPs*) also report that a majority of neurons increase firing rates during motor tasks (Delong, 1972; Mink and Thach, 1991; Turner and Anderson, 1997). These findings are somewhat surprising given the net inhibitory effect on movement expected from increased STN and GPi activity in the classic rate model. The increased firing rates have been interpreted as evidence of the suppression of unwanted or inappropriate movements that might compete with the desired movement (Mink, 1996; Nambu, 2004; Hikosaka et al., 2006), though Turner and Desmurget (2010) cast doubt on this interpretation due to the relatively late onset of activity in GPi relative to movement onset. We will return to this issue in a later section.

Although the primary focus of studies on speech deficits in PD is the basal ganglia, it should be noted that the neurodegenerative and pathophysiological effects of PD impact additional anatomical structures involved in speech motor control, including the cranial nuclei/nerves and cerebral cortex. Sapir (2014) proposed that early manifestations of speech and voice changes in PD may be the result of pathology within the dorsal motor nucleus of the vagus and glossopharyngeal nerves (corresponding to Braak Stage 1–2). Post-mortem studies of PD patients have reported Lewy-type pathology in the peripheral sensory neurons of the upper airway, correlating with dysphagia symptoms (Mu et al., 2013a,b; 2015). Still, it is unclear whether primary pathology in the cranial nerves and their nuclei has a meaningful impact of speech or voice function in PD.

Cortical pathology in PD is observed primarily in the later disease stages (Braak Stage 4–6) and is associated with cognitive decline (Hurtig et al., 2000; Mattila et al., 2000; Kovari et al., 2003; Smith et al., 2019); however, it is unknown whether cortical pathology contributes to PD speech symptoms. Linking speech changes to cortical pathology is especially challenging since definitive measures of brain pathology can only be collected post-mortem. Using non-invasive structural brain imaging, researchers have demonstrated multiple regions of cortical thinning in PD associated with disease progression (Zarei et al., 2013; Tremblay et al., 2021) and poorer cognitive function (e.g., Hanganu et al., 2013; Laansma et al., 2021). Applying this approach to the study of speech in PD, a study by Chen et al. (2020) found that the severity of hypokinetic dysarthria in PD (measured by the voice handicap index) was associated with cortical thinning in the right precentral gyrus and right fusiform cortex, suggesting a possible role of cortical atrophy in the presentation of PD speech symptoms. However, more research is needed to establish whether speech changes in PD can be linked to pathology in cerebral cortex, and it is important to bear in mind that pathological *functioning* in cortex can arise even in healthy cortical tissue due to impaired input from the thalamus and/or other brain regions, further complicating the issue.

## 2 The DIVA/GODIVA neurocomputational modeling framework

**Figure 2:**
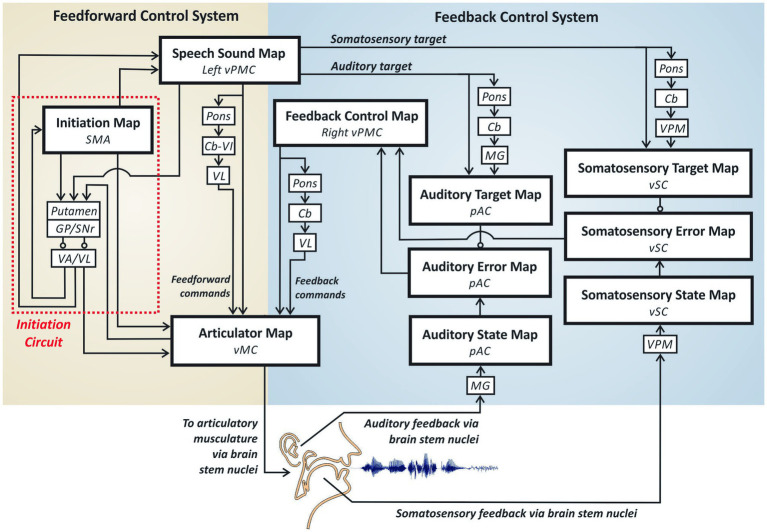
The DIVA model of speech motor control.

Figure 2 provides a schematic of the Directions Into Velocities of Articulators (DIVA) model of speech motor control (e.g., Guenther et al., 2006; Guenther, 2016), which provides a neurocomputational account of the brain mechanisms involved in producing single words. The large boxes in the model each represent a different *cortical map* containing a set of model neurons (*nodes*), with each node given a precise anatomical location (specified in Montreal Neurological Institute stereotactic coordinates). The smaller boxes represent subcortical regions, and arrows indicate excitatory projections while lines with circular heads indicate inhibitory axonal projections. The DIVA model is defined mathematically, and all model components are given precise locations in a standard stereotactic space. The current article will not address the mathematical formulation of the model; we refer the interested reader to the cited papers above for further details.

DIVA divides speech motor control into three sub-processes carried out by largely distinct neural regions: auditory feedback control, somatosensory feedback control, and feedforward control. *The feedforward control subsystem is the most important of the three subsystems for fluent speech production* as it generates the predominant portion of the motor outflow commands. Feedback control mechanisms play only a minor role in the control of an ongoing movement by producing small corrections to errors detected through audition and somatic sensation; the more substantial role for feedback control mechanisms is in helping tune the feedforward commands for future utterances based on errors detected on the current utterance.

The feedforward control subsystem is responsible for the readout of learned motor commands corresponding to motor chunk/words,^1^ without regard for auditory or somatosensory feedback arising from the movements. According to the DIVA model, feedforward commands are stored in synaptic projections from a *speech sound map* (whose nodes each represent a different word’s motor program) in left ventral premotor cortex (*vPMC*) to bilateral^2^ primary motor cortex (*MC*) both directly and via a loop through the pons, cerebellum (*Cb*) and ventrolateral nucleus of the thalamus (*VL*). Together, these regions constitute the *articulation circuit* portion of the feedforward control system, so-named because it is responsible for generating the highly coordinated muscle commands that are required for fluent speech.

The second component of the feedforward control subsystem, the *initiation circuit*, (indicated by red dotted outline in Figure 2) heavily involves the supplementary motor area (*SMA*) and BG—including the striatum, globus pallidus (*GP*), and sub-thalamic nucleus (*STN*) within the cortico-BG motor loop. The nodes in this network do not project directly to the motor periphery; instead, nodes in the SMA’s initiation map effectively determine which motor programs get generated by activating the corresponding nodes in the articulation circuit.

The auditory feedback control subsystem is responsible for detecting and correcting differences between the desired auditory signal for a speech sound and the current auditory feedback. According to the DIVA model, speech sound map nodes project to an *auditory target map* in the higher-order auditory cortical areas of the posterior superior temporal gyrus (*pSTG*) via both cortico-cortical projections and a cortico-cerebellar loop involving the pons, Cb, and medial geniculate (*MG*) nucleus of the thalamus. These projections encode the expected auditory signal for the speech sound currently being produced. Activity in the auditory target map thus represents the auditory feedback that should arise when the speaker hears himself/herself producing the current sound. The auditory target for the current sound is compared to incoming auditory information from the auditory periphery; this information projects to cortex via MG and is represented in the model’s *auditory state map*. If the current auditory feedback is outside the target region, *auditory error map* nodes become active. Like the auditory target map, the auditory state and error maps are hypothesized to lie in pSTG. Auditory error map activities are transformed into corrective motor commands through projections from the auditory error map nodes to the feedback control map in right vPMC, which in turn projects to the articulator map in vMC both directly and via a loop through the pons, Cb, and VL. Notably, the BG are not part of the auditory feedback control circuit in the DIVA model, in keeping with findings that eliminating BG output by inactivating GPi has no impact on feedback control mechanisms in non-human primates (e.g., Desmurget and Turner, 2008).

The DIVA model posits a somatosensory feedback control subsystem operating alongside the auditory feedback control subsystem described above. The main components of the somatosensory feedback control subsystem are hypothesized to reside in ventral somatosensory cortex (*vSC*), including the ventral postcentral gyrus and the supramarginal gyrus. Projections from the speech sound map to the *somatosensory target map*, including cortico-cortical as well as cortico-cerebellar loop projections via the ventral posterior medial (*VPM*) nucleus of the thalamus, encode the expected somatosensory feedback (i.e., tactile and proprioceptive feedback arising from mechanoreceptors and muscle spindles in the vocal tract) during sound production. The model’s *somatosensory state map* represents tactile and proprioceptive feedback from the speech articulators, which arrives from cranial nerve nuclei in the brain stem via VPM. Nodes in the somatosensory error map become active during speech if the speaker’s somatosensory state deviates from the somatosensory target region for the sound being produced. The output of the somatosensory error map then propagates to the feedback control map in right vPMC to transform somatosensory errors into motor commands that correct those errors. As with auditory feedback control, the somatosensory feedback control circuit does not involve BG and thus is not expected to be substantially impaired in PD.

**Figure 3:**
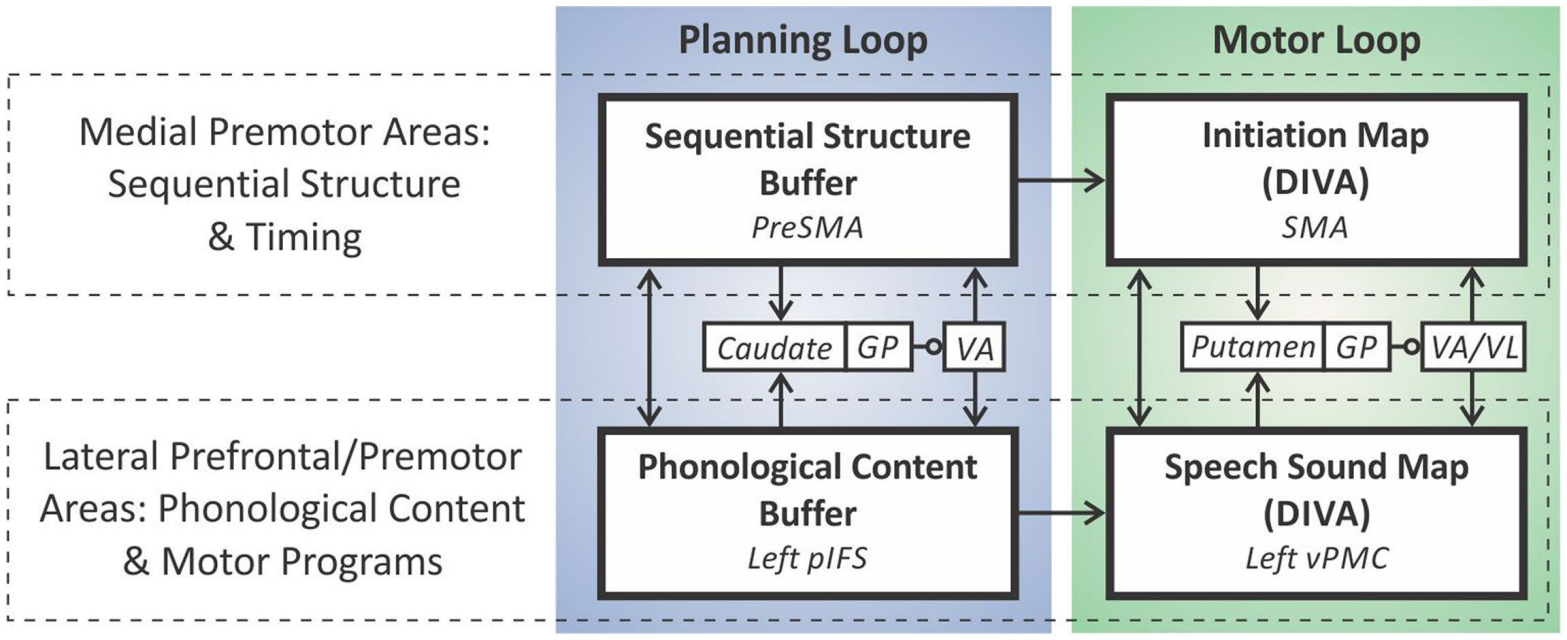
The GODIVA model of speech sequencing.

The GODIVA model (Figure 3) extends the DIVA model by adding two prefrontal cortical regions hypothesized to contain working memory (*WM*) representations of the words in multiple word utterances, such as a phrase or sentence—*preSMA* and the left posterior inferior frontal sulcus (*pIFS*)—as well as basal ganglia and thalamic components of the model’s cortico-BG *planning loop*. Briefly, the phonological material (words) for the utterance is stored in a phonological content buffer in left pIFS; the proper ordering of these items is maintained through interactions (both direct and via the cortico-BG planning loop) between nodes representing rank order in pre-SMA and the corresponding phonological items in pIFS.

*Within the DIVA/GODIVA framework, speech impairments in PD primarily arise from impairment to the initiation circuit of the feedforward control subsystem*, as we will detail in the following section. Subsequent sections will address data bearing on whether additional control mechanisms, beyond the initiation circuit, are impaired in PD. Further detail regarding the DIVA/GODIVA models will be provided as needed in these sections.

## 3 Impaired performance in the initiation circuit

**Figure 4:**
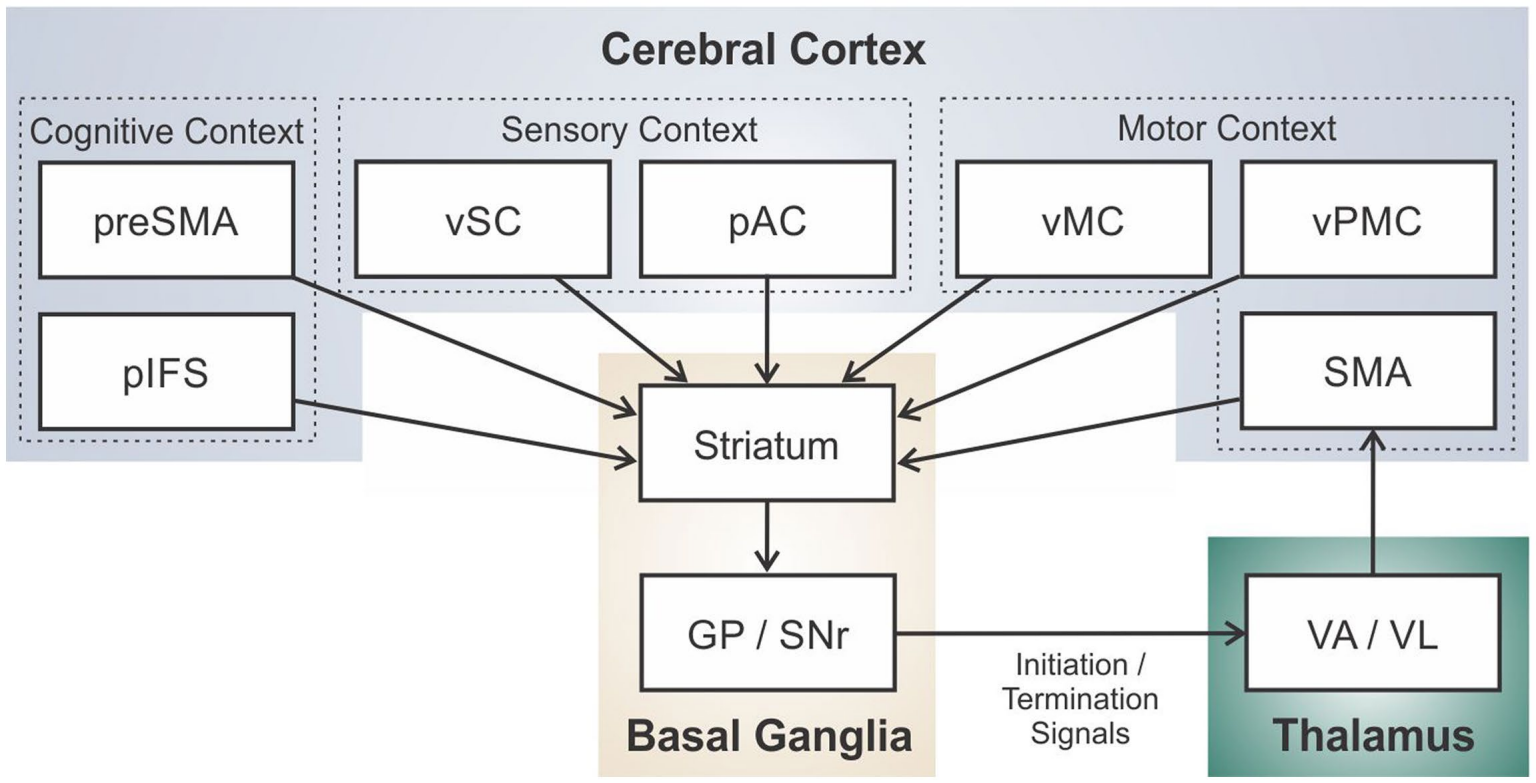
Schematic of the full cortico-basal ganglia-thalamo-cortical loop for speech sequencing and initiation in the DIVA/GODIVA framework.

Figure 4 schematizes the brain regions and connections in the DIVA/GODIVA framework (planning and motor loops combined).^3^ It is helpful to think of this circuit as an *adaptive pattern recognizer* that monitors the current cognitive, sensory, and motor context as represented in cerebral cortex.^4^ For example, if a talker is in the midst of saying the first word in the utterance “test set,” the cognitive context indicates that the current word is “test” and the next word is “set,” the sensory context consists of incoming sensory feedback (filtered according to motor expectations) from the auditory and somatosensory systems, and the motor context consists of the currently active motor cortical neurons sending motor commands to the periphery. When the proper context for releasing the word “set” is recognized (i.e., the current motor commands and sensory feedback indicate the end of the word “test”), the loop effectively terminates the current motor program and initiates the motor program for “set” by deactivating the “test” node in the SMA initiation map and activating the “set” node.

There is substantial evidence that the BG participate actively in learning of a new motor sequence, but as the sequence becomes well-learned, the sequence becomes encoded elsewhere in the brain, e.g., in cortico-cortical projections, without the need for BG involvement in sequence readout (see Turner and Desmurget, 2010 for a review). Prior versions of the DIVA and GODIVA models have not addressed this transfer of performance from BG to cortex; modification of the model to address these findings is ongoing.

GPi inactivation studies indicate that the BG do maintain a role in movement *gain* (or *vigor*^5^) even after the sequence is learned; inactivation of GPi results in a general decrease in movement amplitudes, as commonly seen in PD (e.g., Desmurget and Turner, 2008). In the DIVA model this gain is embodied by a GO signal which multiplicatively scales movement amplitude (cf. Bullock and Grossberg, 1988).

Within our modeling framework, one major functional implication of dopaminergic depletion is a net decrease in excitatory support for the current word’s motor program in the SMA initiation map due to an imbalance in the direct and indirect pathways in which the anti-kinetic indirect pathway dominates. In DIVA, production of the current word starts with activation of the word’s initiation map node and continues as long as that node remains active. Initiation map nodes for upcoming words in the utterance are competing with the current word’s node through inhibitory interactions within SMA. The cortico-BG motor loop is posited to bias this competition in favor of the current word until the sensory-motor context indicating completion of this word is “recognized” by the striatum, at which time the excitatory support for the current word’s initiation map node via the cortico-BG motor loop ends, causing the node to lose the competition to the next word’s initiation map node.

According to this view, reduced support for the current motor program’s initiation map node via the cortico-BG motor loop as a result of PD should have at least two effects. First, the initiation map node for the current word will be less active, thereby producing movements with less gain (long known to be a prominent feature of movements in PD, termed *hypokinesia*) and, in extreme cases, inability to initiate movement. Second, the current word’s node will be more susceptible to competition from initiation map nodes for upcoming words in the utterance, possibly causing it to prematurely “lose out” to the next word, thereby truncating production of the current word. A possible third effect, erroneous selection of the proper motor program amongst competing alternatives (cf. Mink, 1996), would result in improperly ordered movement sequences. However, speech sequencing errors of this type are not characteristic of disorders of the BG such as PD (Duffy, 2019; Camerino et al., 2022) nor after pallidotomy (e.g., Green et al., 2002; Troster et al., 2003), indicating that the order of individual movements in well-learned sequences is represented elsewhere, e.g., in cortico-cortical connections. We will return to this topic in a later section.

**Figure 5:**
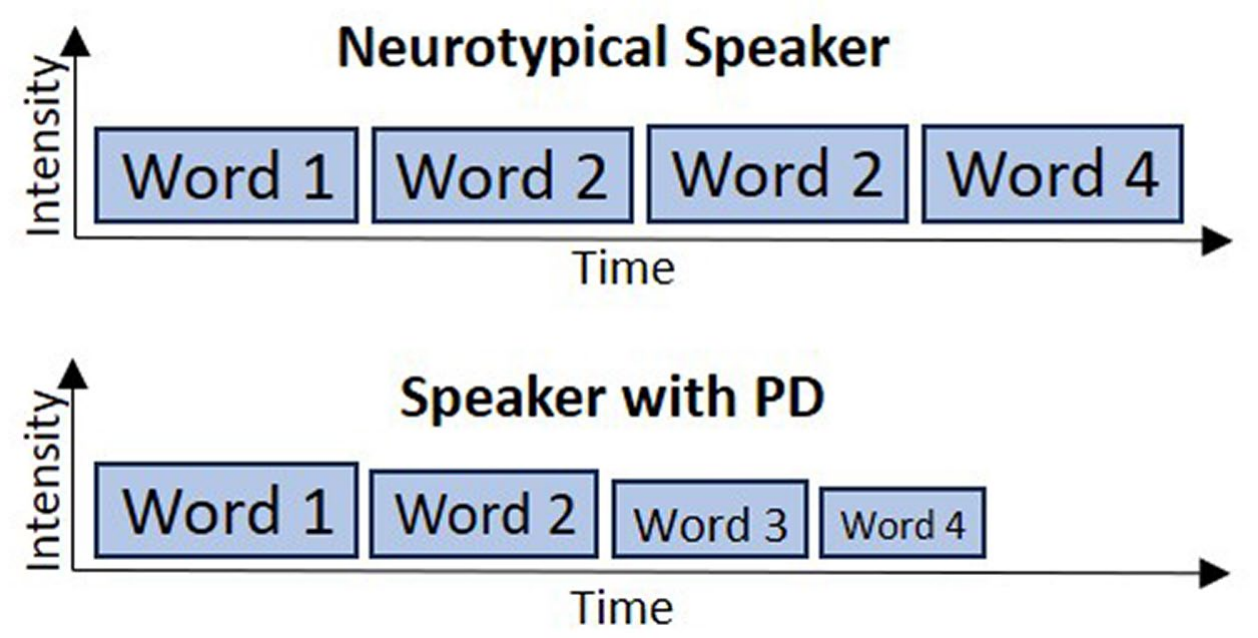
Schematic of accelerating speech resulting from an impaired ability to maintain activation of ongoing motor programs’ representations in the initiation map.

Figure 5 provides a schematized depiction of the situation when producing a four-word utterance, with the motor program for each word represented with a box. The top plot shows the intensity (box height) and duration (box width) for each word in the 4-word utterance for a neurotypical speaker, while the bottom row depicts a speaker with PD. The first word is produced with normal intensity and duration by the PD speaker since it does not depend on the cortico-BG motor loop for activation of the SMA initiation map node for this word. However, for subsequent words, decreased support from the cortico-BG motor loop leads to decreased movement intensity as well as premature motor program termination. A further hypothesis—not directly derived from the GODIVA model but motivated by findings described below—is that, in speakers with PD, the reduced support from the cortico-BG motor loop gets progressively worse across the production of extended speech utterances – similar to the common observation in PD of progressively smaller handwriting as a patient continues to write (*micrographia*; McLennan et al., 1972). Note that, in this account, the overall speaking rate (measured in number of words, syllables or phonemes per unit time), can actually be *faster* in PD than in controls; experimental studies have produced mixed results, with some showing faster rates in PD (Skodda and Schlegel, 2008), others showing slower rates (Dworkin and Aronson, 1986; Ludlow et al., 1987), and still others showing no differences (Lowit et al., 2006).^6^ Despite variable reports of speaking rate, one common feature in PD is the *acceleration* of speech rate (i.e., increased speech rate over time; Tjaden, 2000; Moreau et al., 2007; Skodda and Schlegel, 2008; Skodda et al., 2010; Flasskamp et al., 2012; Rusz et al., 2015). This acceleration of speech may be due to an impaired ability to keep motor programs active that worsens over the course of a long utterance, as schematized in Figure 5.

A number of additional behavioral observations concerning speech in PD support this account. (Table 1 provides a summary of experimental findings described in this and subsequent sections.) Reduced movement intensity/scaling is a hallmark of speech deficits in PD, including reduced vocal intensity (*hypophonia*) and reduced articulatory movement extent (*hypokinetic dysarthria*; Sapir, 2014; Duffy, 2019). In line with our theoretical view, it has been hypothesized that reduced scaling of speech movements results, at least in part, from impaired internal cueing mechanisms (Sapir, 2014), which are mediated by preparatory activity in SMA (Mushiake et al., 1991; Halsband et al., 1994). In fact, one key goal of the Lee-Silverman Voice Treatment (LSVT LOUD®) is to recalibrate internal cueing mechanisms for voice amplitude scaling through intensive voice therapy (Ramig et al., 2011). Although our theoretical view attributes this reduced intensity to reduced support of SMA initiation map nodes from the cortico-BG motor loop, alternative accounts have been put forward. For example, it has been hypothesized that decreased movement gain in PD results from increased rigidity of the speech musculature (Hunker et al., 1982; Pellat et al., 1983; Hanson et al., 1984; Gallena et al., 2001) and respiratory musculature (Solomon and Hixon, 1993; Sadagopan and Huber, 2007). However, a causative role of rigidity on reduced amplitude scaling has been called into question by others (Netsell et al., 1975; Caligiuri, 1987; Connor and Abbs, 1991; Sapir, 2014).

**Table 1:** Summary of findings concerning speech production in PD patients.

Subsystem	Competency	Finding	Citations
Feedforward control	Speech initiation	Reduced speech intensity compared to controls	Duffy (2019) (review); Sapir (2014) (review)
		Reduced emphatic stress compared to controls	Darley et al. (1969a,b); Pell et al. (2006)
		Acceleration of speaking rate	Flasskamp et al. (2012); Skodda and Schlegel (2008); Skodda et al. (2011); Tjaden (2000); Moreau et al. (2007); Rusz et al. (2015)
	Speech sequencing	Impaired learning or retention of new speech sequences compared to controls	Kaipa et al. (2016); Schulz et al. (2000); Whitfield and Goberman (2017)
Auditory FB control	Pure auditory processing	Impaired F1 discrimination compared to controls	Mollaei et al. (2019)
		Impaired loudness and duration detection	De Groote et al. (2020) (review)
		Impaired prosody perception	Lloyd (1999); Lima et al. (2013)
		Anomalous transient-evoked otoacoustic emissions	Di Mauro et al. (2017); De Keyser et al. (2019)
		Worsening of age-related hearing loss compared to controls	Folmer et al. (2017); Pisani et al. (2015); Scarpa et al. (2020); Shalash et al. (2017); Vitale et al. (2012); Vitale et al. (2016); Yylmaz et al. (2009)
	Audio-motor processing	Better pitch discrimination while vocalizing compared to controls	Mollaei et al. (2019)
		Reduced pitch and F2 adaptation compared to controls	Mollaei et al. (2013); Abur et al. (2018)
		Normal pitch and formant adaptation on meds	Abur et al. (2021)
		Normal reflexive perturbation responses on meds	Abur et al. (2021)
		Exaggerated pitch reflexive responses to unpredictable perturbations off meds	Huang et al. (2019); Mollaei et al. (2016, 2019); Chen et al. (2013); Liu et al. (2012); Huang et al. (2016)
		Normalization of exaggerated pitch reflexive responses with intervention	Behroozmand et al. (2019); Li et al. (2021)
		Reduced formant reflexive perturbation responses off medication	Mollaei et al. (2016, 2019)
Somato. FB control	Pure somato. processing	Worse orofacial somatosensation	Schneider et al. (1986); Hammer (2009); Hammer and Barlow (2010); Hammer et al. (2013); Chen and Watson (2017)
	Somato-motor processing	Anomalous responses to mechanical perturbations	Alfonsi et al. (1993); Caligiuri and Abbs (1987); Mefferd and Bissmeyer (2016)
		Blunted perception of respiratory load when breathing	Hegland et al. (2019)

This table necessarily simplifies study findings in order to organize a highly varied literature; please consult the original studies for additional details.

Additional support for this theoretical view can be found in the neuroscience literature. A common finding in single unit studies of GPi in non-human primates is that GPi neurons are typically sensitive to specific characteristics of a movement, such that different neurons are active in different motor contexts (Turner and Anderson, 2005); this finding accords well with the idea of the cortico-BG loop as a pattern recognizer. Activity of many GPi neurons scales monotonically with movement gain/peak velocity/force (Turner and Anderson, 1997), as represented by the GO signal in the DIVA model; this property has also been found in functional neuroimaging studies of human limb movements (Turner et al., 2003; Desmurget et al., 2004; Spraker et al., 2007; Thobois et al., 2007; Wasson et al., 2010).

Neuroimaging studies of speech production in PD have reported conflicting findings concerning the level of activation in SMA for PD patients compared to controls, with some studies reporting hypoactivation of SMA (Baumann et al., 2018; Narayana et al., 2020), some reporting hyperactivation of SMA (Liotti et al., 2003; Pinto et al., 2004; Rektorova et al., 2007), and others reporting no group differences in SMA activity between PD and controls (Pinto et al., 2011; Arnold et al., 2014; Klobusiakova et al., 2021; Manes et al., 2023). Regarding voice intensity, Liotti et al. (2003) found no differences in BG or SMA activity when comparing loud versus habitual overt speech in participants with PD. In contrast, a study of PD and HC participants performing covert (silent) speech in “loud” (as if shouting on a windy beach) and normal conditions found that SMA was hypoactive for PD participants compared to controls in the normal intensity condition but not the “loud” condition prior to voice therapy; group differences in SMA activity were no longer present after voice therapy (Baumann et al., 2018). Hypoactivity of right premotor cortex, but not SMA, was found during sustained phonation in PD participants with hypophonia compared to older healthy controls, suggesting that right premotor cortex may be linked to phonatory drive in PD (Manes et al., 2023). The discrepancy of findings between neuroimaging studies may be in part due to the limited temporal resolution of fMRI, the type of speech task employed (e.g., overt vs. covert speech), presentation of speech symptoms, and medication state. Taken as a whole, however, the existing literature suggests that overall SMA activation may not differ substantially between PD and controls during motor acts such as speech; in terms of our theoretical framework, this could occur because decreased activation of the correct SMA node is offset by increased activation in other (possibly competing) nodes, e.g., due to reduced inhibition from the chosen node, thereby resulting in little or no net activity as measured by fMRI.

Although many findings are in accord with our account, a few commonly reported experimental findings pose difficulties for the DIVA/GODIVA account of BG involvement in initiating speech. As noted in Section 1, activity in STN (Georgopoulos et al., 1983; Wichmann et al., 1994) and GPi (Mitchell et al., 1987; Turner and Anderson, 1997; Doyon et al., 2009; Jin and Costa, 2015) increases during peri-movement periods, which according to the classic rate model should inhibit movement in cortex, whereas in DIVA the BG facilitate production of the current motor program. A possible explanation for this is that the bulk of BG output acts to inhibit movements other than the correct movement such that the decrease in inhibition for the correct movement is outweighed (on average) by the increased inhibition of other movements (Mink, 1996; Nambu, 2004). Another possibility is that it is the *temporal pattern* of BG output that is crucial rather than the overall amount of activity (e.g., Levy et al., 2000; Brown, 2003). Within this view, a pathological increase in synchronized oscillatory firing between the basal ganglia nuclei and their output structures in PD can interfere with the ability to reliably transmit thalamic signals to cortex.

Perhaps a more troubling issue for the DIVA/GODIVA view is the frequently noted finding in the NHP literature that, for well-learned movements, activation in striatum and GPi typically lags activity in cortex and thalamus at movement onset in reaction time experiments, and furthermore reaction times are not negatively impacted by GPi inactivation in NHPs nor in PD pallidotomy recipients (Schwab et al., 2020; see Turner and Desmurget, 2010, for a review). On the surface these findings seem to contradict with the DIVA model’s inclusion of a role in movement initiation for the BG, at least for well-learned words produced in isolation.

To further consider this issue, it is helpful to recognize that speech consists of (at least) two levels of sequencing: (1) sequencing of phonemes within well-learned words (whose production can be considered “overlearned”), and (2) sequencing of words in larger utterances which are not typically well-learned since each utterance can contain a unique combination of words. The later mean onset of GPi activity compared to cortical activity found in reaction time experiments as described above suggests that the initiation of a well-learned word in isolation may not require BG involvement; however, Lipski et al. (2018) report that a subset of units in STN fire well before speech onset, leaving open the possibility of BG involvement in utterance initiation. Furthermore, even if initiation of the first phoneme in a well-learned word is primarily carried out by cortex, transitions from the first to subsequent phonemes might still involve the BG, which is ideally situated to detect the cognitive, sensory, and motor context indicating the completion of an ongoing movement given its widespread input from the relevant cortical areas. Such a role (if it exists) would appear to be supplementary rather than central given that well-learned sequences can be produced without error after GPi inactivation or pallidotomy (discussed further in Section 6.3), but the BG may contribute under more demanding conditions such as fast speech or when producing long or infrequent words that are not overlearned. Further study is needed to address these issues.

Although a central role for the BG in single word production by mature speakers is questionable, the BG very likely play a role in speech motor learning. Prior electrophysiological studies have indicated a role for the BG in a wide range of motor learning tasks (e.g., Miyachi et al., 1997; Sage et al., 2003; Ashby et al., 2010; Piron et al., 2016), and pallidotomy has been shown to negatively impact the learning of new motor sequences (Brown et al., 2003; Obeso et al., 2009). Turner and Desmurget (2010) posit that the BG are heavily involved in early learning stages, particularly for reward-based tasks (with learning modulated by phasic striatal dopamine training signals), based in part on findings that the striatum exhibits rapid changes in an associative learning task, whereas changes in cortex occur on a much slower time scale (Pasupathy and Miller, 2005). With regard to speech in PD, this view predicts that individuals with PD will be impaired in learning to speak a second language, or learning to produce words that are new to them in their native language(s); these hypotheses are supported by a several studies on speech motor learning in PD patients (Schulz et al., 2000; Kaipa et al., 2016; Whitfield and Goberman, 2017). Collectively, the literature strongly suggests that the BG are involved in word learning during speech development as well as learning of new words later in life.

There are also few studies we are aware of directly bearing on the question of whether the initiation of words within a larger utterance, such as a sentence or phrase, critically involves the cortico-BG loop, though Lipski et al. (2023) report speech sequence-related activity in STN in a task involving production of consonant-vowel syllable triplets. Given that naturally occurring speech does not typically involve repeated production of the same sentence nor over-learning of a given word sequence (except possibly for very commonly used sequences, such as one’s phone number), it seems unlikely that a reward-based learning mechanism would be necessary for word initiation in conversational speech, but experimental study of this possibility is still needed before a strong conclusion can be drawn. Turner and Desmurget (2010) also propose a role for the BG in adjusting movement vigor based on motivational factors such as reward contingencies. One can imagine a role for such a mechanism in emphatic stress during running speech if one considers the desire to emphasize a particular word a form of motivational signal; indeed, reduced stress is an established feature of dysarthria in PD (Darley et al., 1969a,b; Pell et al., 2006).

## 4 Audiomotor impairments in PD

The degree to which PD impacts auditory processing is unclear. In line with non-human primate studies showing responses in STN and GPi to passive movements (Delong et al., 1985; Wichmann et al., 1994), pure auditory responses have been reported in the STN during speech (Tankus and Fried, 2019; Tankus et al., 2021). Unpublished data collected during deep brain stimulation surgery from our group support this idea; STN neurons respond to auditory speech stimuli and even show selectivity for certain speech sounds, suggesting a possible role in sensorimotor processes.

Although some studies have found no difference in pure auditory perceptual task performance between PD patients and age-matched controls (e.g., Di Mauro et al., 2017; De Keyser et al., 2019) others have found a worsening of age-related high-frequency hearing loss in PD (Yylmaz et al., 2009; Vitale et al., 2012; Pisani et al., 2015; Vitale et al., 2016; Folmer et al., 2017; Shalash et al., 2017; Scarpa et al., 2020). With respect to speech, people with PD tend to perform comparably to older healthy controls during phoneme discrimination tasks (Lloyd, 1999; Graber et al., 2002; Ravizza, 2003; Lima et al., 2013); however, impairments in prosodic processing have been noted by Lloyd (1999) and Lima et al. (2013). Furthermore, there is some evidence for abnormal pitch and formant perception in PD. Mollaei et al. (2019) found that discrimination for first formants was significantly lower in PD compared to age and sex-matched healthy controls during passive listening. Meanwhile, PD participants showed better pitch discrimination compared to controls during active voice production. Based on a comprehensive review of the research literature on auditory deficits in PD, De Groote et al. (2020) concluded that speech and pitch perception appear normal in PD, whereas perception of loudness and duration are impaired, with PD patients overestimating intensity of less intense speech and underestimating intensity of more intense speech. Interestingly, loudness and duration are also impaired in the speech *output* of individuals with PD, suggesting the possibility that impairments in perception of these acoustic parameters may result from an impaired ability to “internally simulate” them. However, the inverse conclusion, that impaired perception leads to impaired production of these parameters, cannot be excluded based on the current evidence.

Within the DIVA/GODIVA framework, the BG do not play a direct role in auditory feedback control. However, impairment of auditory processing would be expected to affect both feedback and feedforward control mechanisms since an impaired ability to detect mismatches between the auditory target and current auditory state would impair both the ability to perform online corrections via feedback control as well as the ability to tune feedforward commands based on auditory feedback. Another possibility is that auditory feedback control may be normal, with only an impairment in updating feedforward commands. These possibilities are discussed in the following paragraphs.

Auditory feedback control of speech in PD has been probed using unexpected auditory perturbations applied to a speaker’s own speech in real-time; such perturbations induce *reflexive* compensatory responses involving auditory cortex and right inferior frontal cortex (Tourville et al., 2008). If the perturbation is consistent rather than unexpected, neurotypical speakers will also adjust their feedforward commands (sensorimotor *adaptation*), as evidenced by after-effects once the perturbation is removed.

Although impaired adaptation in response to sustained perturbations of pitch and F1 have been demonstrated in PD (Mollaei et al., 2013; Abur et al., 2018), reflexive responses to unpredicted perturbations of pitch and loudness appear to be either normal (Abur et al., 2021, who tested participants on medication) or larger than normal (Liu et al., 2012; Chen et al., 2013; Huang et al., 2016; 2019; Mollaei et al., 2016, 2019; these studies tested PD participants off medication). Deep brain stimulation applied to STN has been shown to normalize the pitch shift reflex, implicating the basal ganglia in the abnormally large pitch shift reflex in PD (Behroozmand et al., 2019). The lack of impairment in reflexive responses to unexpected pitch perturbations indicates that individuals with PD properly detect pitch errors (see also Mollaei et al., 2019; Abur and Stepp, 2020) which in turn indicates that impaired auditory processing is not the primary cause of impaired audio-motor adaptation to pitch perturbations in PD. In sum, the literature on pitch perturbation responses in PD is consistent with the lack of a BG component to auditory feedback control in the DIVA/GODIVA framework.

The literature on F1 perturbations in PD is somewhat less clear; whereas Abur et al. (2021) find normal F1 perceptual acuity as well as reflexive and adaptive response to F1 perturbations in PD patients who are on medication, reduced reflexive responses (Mollaei et al., 2016, 2019) and adaptive responses (Mollaei et al., 2013) to F1 perturbations have been demonstrated in PD patients tested while off medication, perhaps due to a reduced ability to discriminate small F1 differences (Mollaei et al., 2019). The finding of reduced F1 reflexive responses in PD poses a challenge to the DIVA/GODIVA framework as it suggests a role of the BG in auditory processing and/or auditory feedback control, neither of which are currently represented in the model. One possible explanation for differences between f0 and F1 responses in PD is that the sensorimotor control of speech involves different mechanisms for the phonatory and articulatory systems. As Mollaei et al. (2016) note, the suprasegmental aspects of speech (e.g., pitch and loudness) are more rapidly affected by changes in hearing status (Svirsky et al., 1992; Lane et al., 1997; Perkell et al., 2007), while phonemic parameters, such as vowel formants, are more resistant to changes in hearing status (Cowie and Douglas-Cowie, 1992; Perkell et al., 1992).

According to the DIVA model, reflexive responses to auditory perturbations are mediated by the auditory feedback control system as follows. Auditory errors induced by the perturbation are detected by an auditory error map in auditory cortex (specifically, bilateral posterior superior temporal gyrus) that compares incoming auditory signals with the auditory target for the current sound. Detected errors are then transmitted to right hemisphere premotor cortical regions, which transform the error into a corrective movement command. The size of this corrective command is scaled by an auditory feedback control gain parameter. Neuroimaging studies of reflexive auditory perturbations have verified the existence of auditory error maps in pSTG (Tourville et al., 2008; Parkinson et al., 2012).

Within the DIVA model, exaggerated reflexive responses can result in several ways. First, an abnormally large auditory feedback control gain would lead to a larger motor response for auditorily perceived errors. Second, since the overall motor command in DIVA is composed of both feedforward and feedback control components, a reduction in the gain of the feedforward control component results in an increased influence of feedback control mechanisms on the overall motor command, which in turn can result in increased reflexive responses to auditory perturbations. Reflexive responses in PD are reduced when the speaker volitionally increases loudness, either as part of therapy (Li et al., 2021) or external cuing (Huang et al., 2019), adding further support for interpreting exaggerated reflexive responses in PD as a secondary consequence of reduced feedforward control gain. A third possible mechanism is a reduction in the somatosensory feedback control gain, which, like a reduction of the feedforward command gain, has the effect of increasing the influence of auditory feedback control mechanisms on the overall motor command. This possibility is supported by the somatosensory deficits in PD described in the next section. Collectively, the observations described in this section suggest that anomalies in auditory feedback control-mediated responses are likely due to impairments in the motor and/or somatosensory systems rather than impairments in auditory processing or auditory feedback control mechanisms *per se*. Consistent with this view, auditory cues can be used to entrain movements in acoustic therapies (Leuk et al., 2020).

The finding of reduced adaptation to sustained auditory perturbations in PD (despite normal or larger-than-normal reflexive responses) is indicative of impaired updating of feedforward commands, captured by a learning rate parameter in the DIVA model. The DIVA model posits a central role for the cerebellum in the updating of feedforward commands, but currently the model does not explicitly implicate the basal ganglia in this process. The findings of impaired sensorimotor adaptation in PD reported here, combined with several reports of reduced adaptation in individuals who stutter (another disorder often associated with basal ganglia malfunction), seem to indicate that dopamine and/or the cortico-BG loop contribute to audio-motor adaptation in a way that is not currently captured by the DIVA/GODIVA framework.

Impaired auditory processing of important acoustic cues such as F1 could also impair the ability of the striatum to identify the proper sensorimotor context for terminating the current motor program and launching the next one in the sequence. This could lead to problems in initiating and maintaining motor programs, as described in Section 3. Some support for this possibility has been found using fMRI-based resting state functional connectivity; Manes et al. (2018) found reduced functional connectivity between left putamen and left superior temporal gyrus in PD participants with speech impairment compared to PD participants with no speech impairment.

## 5 Somatomotor impairments in PD

Motor action and somatic sensation are intimately linked. For example, the gamma motor neuron system uses efferent motor signals to adjust the sensitivity of muscle spindles for detecting changes in muscle length, a process referred to as proprioception. Though not as directly linked to motor activity as proprioception, tactile feedback from mechanoreceptors in the skin also depends heavily on self-generated movement for its interpretation; e.g., contact between the tongue and palate is expected if the tongue muscles have positioned it against the palate, but is indicative of a movement error if it occurs when the tongue has been commanded to a position that should not involve palatal contact. Indeed, somatosensory deficits have been well documented in PD across a number of nonspeech effectors (Konczak et al., 2009; Conte et al., 2013; Lee et al., 2018; Halperin et al., 2021) as well as along the vocal tract, including the airway (Hammer et al., 2013; Troche et al., 2014; Hegland et al., 2019), larynx (Hammer, 2009; Hammer and Barlow, 2010), jaw (Schneider et al., 1986), and tongue (Chen and Watson, 2017). Changes in vocal tract somatosensation have been shown to correlate with functional measures of speech, voice, and swallowing in people with PD. For example, Hammer and Barlow (2010) reported that larynx mechanosensory detection thresholds correlated with voice intensity, respiratory driving pressure, laryngeal resistance, and lung volume expended per syllable, and Chen and Watson (2017) demonstrated that reduced tongue tip acuity in PD was linked to poorer sibilant contrasts.

Signs of impaired somatosensory processing in PD can be found at multiple levels of the nervous system. Diminished neural activity compared to controls in somatosensory cortex of PD patients in response to passively delivered tactile stimulation of the digits has been shown using PET (Boecker et al., 1999) and fMRI (Nelson et al., 2018). Seemingly paradoxically, PD patients commonly show larger-than-normal reflexive responses to tactile or electrical stimulation of the lips (e.g., Caligiuri and Abbs, 1987; Alfonsi et al., 1993). However, these increased reflexive responses are typically interpreted as the result of decreased cortical inhibition of the subcortical reflex loop, thereby accounting for both decreased cortical activity under somatosensory stimulation and increased reflexive responses in PD.

Another technique used to probe somatosensory processing in the brain involves application of transcranial magnetic stimulation (TMS) over motor cortex to induce motor responses (e.g., finger muscle twitches) while tactile stimulation is simultaneously applied (e.g., mechanical stimulation of the finger). Neurotypicals exhibit *afferent inhibition*, evidenced by reduced motor response to TMS in the tactile stimulation condition compared to TMS applied in the absence of tactile stimulation. Several additional studies have demonstrated reduced afferent inhibition in PD compared to neurotypical controls (Sailer et al., 2003; Tamburin et al., 2003; Nelson et al., 2018); furthermore, Sailer et al. (2007) report improved afferent inhibition in PD patients when DBS stimulation is on compared to when it is off.

Conte et al. (2013) posit that dopaminergic denervation of the BG in PD may result in decreased response specificity of incoming sensory information. Neurophysiological studies in animal models have shown distinct representations of effectors in the STN and GP during passive movements (i.e., neurons responding selectively to movement of a single upper or lower limb joint); however, after selective lesions of the SNc are used to induce a parkinsonian state, the effector representation becomes less distinct—activity becomes spatially distributed throughout larger portions of the nuclei and neurons more frequently respond to passive movements of multiple joints (Filion et al., 1988; Boraud et al., 2000). Extrapolating to speech production in PD, it is possible that deficient somatosensory function of the vocal tract is the result of blurred sensory representations of speech effectors within the motor system (cf. Nambu, 2011).

An additional possible source of speech-related somatosensory deficits is neuropathology within the peripheral nerve fibers. Mu et al. (2013b, 2015) conducted post-mortem analyses of sensory nerves in the pharynx and upper airway and found evidence of Lewy-type pathology in people with PD. Furthermore, PD participants with dysphagia had more markers of pathology in regions critical for swallowing reflexes (Mu et al., 2013b, 2015). To date, no studies have investigated whether sensory nerve pathology along the vocal tract correlates with voice or speech symptoms in PD. However, the observations of Lewy-type pathology within the sensory nerves of the upper airway suggest that peripheral sensory mechanisms of voice and speech dysfunction merit further investigation.

In the DIVA/GODIVA framework, the BG are not directly involved in somatosensory feedback control of speech movements. However, the somatosensory feedback controller depends on accurate somatosensory feedback for proper operations, and deficits in somatosensory feedback processing will thus impact somatosensory feedback control (e.g., responses to externally applied forces). The larger-than-normal reflexive responses to tactile stimulation suggest an increased gain of the somatosensory feedback control system in PD, possibly due (at least in part) to reduced cortical inhibition of subcortical feedback loops, or to reduced “competition” with feedforward commands as posited above for exaggerated reflexive responses to auditory perturbations. It is also possible that these two accounts are two sides of the same coin; i.e., reduced cortical inhibition of subcortical feedback loops may be (part of) the mechanism that reduces the influence of feedforward commands on the somatosensory feedback controller’s responses to perturbations.

An alternative interpretation of increased reflexive responses in PD is that the brain may be “under-sensing” the size of the corrective responses, perhaps due to impaired proprioception. This view is compatible with the findings of decreased cortical activity in response to tactile stimulation noted above, and it also accords with the finding of Mefferd and Bissmeyer (2016), who noted an anomalous increase in vowel contrasts in PD when speaking with a bite block. In the absence of any compensation, the blocking of jaw movement imposed by a bite block would have the effect of reducing vowel contrasts. Neurotypical speakers compensate by increasing tongue and lip movements just enough to overcome the jaw-limiting effect of the bite block. In contrast, speakers with PD continue the compensatory movements beyond their normal productions, thereby producing larger vowel contrasts than they produce in the absence of the bite block. In the DIVA model, an increase in the gain of the somatosensory feedback controller would speed up compensatory movements, but these movements would not overshoot the targets for normal (unperturbed) speech; a sustained overshoot would only occur if the system was underestimating the effects of the compensatory movements generated by the somatosensory feedback control system.

## 6 Effects of pharmacological and surgical treatments for PD on speech

### 6.1 Pharmacological treatments

In PD, the primary medical approach is to correct for dopaminergic deficiencies using dopamine replacement therapy (DRT; i.e., levodopa) or dopamine agonists. DRT significantly improves clinical motor signs of PD (Katzenschlager and Lees, 2002). Studies of oral motor function suggest that levodopa may improve the strength (Cahill et al., 1998; De Letter et al., 2003; Lechien et al., 2019) and physiology (Robertson and Hammerstad, 1996; Gallena et al., 2001; Tawadros et al., 2012) of the speech articulators. However, the effects of levodopa on speech production are less straightforward.

Despite providing significant clinical improvement for motor signs in PD, DRT appears to have limited efficacy for improving functional speech outcomes. The majority of studies examining the acute effects of dopaminergic therapy found no significant group-level treatment effects on measures of speech and voice function (Kompoliti et al., 2000; Ho et al., 2008; Plowman-Prine et al., 2009; Skodda et al., 2010, 2011; Fabbri et al., 2017; Whitfield et al., 2018; Cavallieri et al., 2021; Tykalova et al., 2022). However, some studies have reported levodopa-related improvements in acoustic measures of vocal pitch and quality (Sanabria et al., 2001; Lechien et al., 2019; Pah et al., 2021). Ho et al. (2008) reported a trend towards improved vocal intensity when comparing medication ON and OFF states. Improvements in voice onset times (Fischer and Goberman, 2010), percent pause times (Goberman et al., 2005) and intelligibility (De Letter et al., 2005, 2007) have also been reported. However, in general the effects of dopaminergic medication on speech outcome measures do not appear to be robust or consistent across studies.

The inconsistency of dopaminergic effects on speech outcomes may be in part related to the variability of speech characteristics among participants with PD. While the evidence for a generalized effect of DRT on speech is limited, some studies have noted treatment related improvements for individuals with specific speech profiles as well as groups characterized by more severe speech characteristics. For example, Skodda et al. (2010) reported that a subset of participants with vowel articulation difficulties experienced a significant improvement in vowel articulation following DRT. Within the phonatory system, Cushnie-Sparrow et al. (2018) found that DRT improved voice quality for a subset of participants who had poor voice quality in the off-medication state. Gallena et al. (2001) found that levodopa helped to reduce overactive laryngeal EMG activity for individual participants and noted that these changes correlated with improved speech outcomes. Im et al. (2019) found that PD participants who were categorized as having dysfluencies had a significant effect of DRT for improving fluency. Interestingly, Cushnie-Sparrow et al. (2018), Gallena et al. (2001), and Im et al. (2019) found that levodopa responsiveness was associated with greater symptom severity. Using a longitudinal approach, Rusz et al. (2021) investigated the long-term effects of DRT on speech outcomes for *de novo* PD participants with different subtypes of speech characteristics—phonatory-prosodic, articulatory-prosodic, and prosodic. The study showed that after 1-year of DRT overall speech impairment improved in the phonatory-prosodic group and remained stable in the articulatory-prosodic and prosodic groups, while those who did not initiate DRT showed a decline in speech function. Taken together, these studies suggest that DRT can improve speech symptoms in those with significant speech impairment, while also highlighting the need to better understand how DRT affects the speech of PD patients with different speech profiles and different levels of speech severity.

Within the DIVA/GODIVA framework, dopaminergic depletion results in a net decrease in excitatory support for feedforward control processes in the SMA initiation map, speech sound map, and articulator map. Restoring dopaminergic input to the striatum via pharmacological treatment could alter speech sensorimotor control in a few ways. First, increasing excitatory support for the SMA initiation map would help facilitate the initiation of speech motor programs in PD and would allow the speaker to produce speech movements with higher gain. Second, restoring dopaminergic input to the striatum allows the initiation map to more effectively monitor the correct sensorimotor context for initiating the next motor program in a sequence. In doing so, dopaminergic therapy would help to strengthen the activation of the current motor program while reducing competition with subsequent motor programs. This could reduce the truncation and scaling decay of subsequent words in a sequence, thereby reducing the accelerated speech rate that is seen in PD. Third, the therapeutic effects of dopamine replacement on feedforward control would reduce the need for a compensatory reliance on auditory feedback. This view posits that PD patients who are off-medication state rely more heavily on sensory feedback control to compensate for feedforward control deficits. Meanwhile, patients in the on-medication state may rely less on sensory mechanisms due to the positive effects of levodopa on feedforward motor control. As a result, we would expect over-exaggerated compensatory responses to reflexive pitch perturbations in the off-medication state (Liu et al., 2012; Chen et al., 2013; Huang et al., 2016; Mollaei et al., 2016, 2019), but not in the on-medication state (Abur et al., 2021), consistent with the existing literature on auditory perturbation responses in PD.

### 6.2 Deep brain stimulation

Deep brain stimulation (DBS) is the gold-standard treatment for moderate- to late-stage PD for which motor symptoms are not well-controlled by DRT. DBS for PD delivers electrical impulses to either the STN or GPi,^7^ providing relief from the most debilitating PD motor symptoms (Starr et al., 1998). DBS induces physiological changes at multiple spatial (e.g., cellular, microcircuit, and network) and temporal (milliseconds to years) scales (Neumann et al., 2023). Although its precise therapeutic mechanisms remain unknown, there is a growing consensus that DBS works by dampening transmission of the pathologic patterns of neuronal activity associated with PD (synchronized oscillatory spiking) while not affecting the overall firing rate of BG output neurons (Bar-Gad et al., 2004; Agnesi et al., 2013; Rosenbaum et al., 2014; Wichmann et al., 2018; Neumann et al., 2023). Despite DBS’s efficacy for some limb motor symptoms (tremor and rigidity), speech deterioration is a concern for patients considering DBS for movement disorders (Bronstein et al., 2011; Aldridge et al., 2016). Because recent comprehensive reviews of DBS’s effects on speech have already been conducted (Skodda, 2012; Aldridge et al., 2016; Baudouin et al., 2023), we will only summarize the themes here and comment on what we can infer about healthy speech and PD speech from studies of DBS.

Using DBS outcome studies as a circuit dissection tool to understand the role of the BG in speech can be problematic. In addition to which nucleus is targeted, the following factors must be considered: behavioral measure (acoustic vs. intelligibility vs. self-perception outcomes); medication state (OFF vs. ON); electrode location within the target nucleus (e.g., anterior vs. posterior STN); stimulation parameters (low vs. high intensity and frequency); volume of neural tissue affected by the electrode current; and timescale of effects (immediate vs. gradual). The heterogeneity of behavioral measures across studies alone makes interpreting the effects of DBS challenging; some studies report acoustic parameters (D'Alatri et al., 2008; Van Lancker Sidtis et al., 2010; Karlsson et al., 2013; Martel-Sauvageau and Tjaden, 2017) while others focus on intelligibility ratings (Tornqvist et al., 2005; Tripoliti et al., 2011; Chiu et al., 2020) or self-perceived speech ratings (Miller et al., 2006; Wertheimer et al., 2014; Kopf et al., 2022). Furthermore, two different time windows of effects of stimulation should not be conflated: (a) acute intraoperative microelectrode stimulation mapping or DBS lead stimulation after it’s turned on in the weeks after surgery, and (b) long-term (months to years) cumulative effects of brain stimulation. Distinguishing between these time windows has important implications for the interpretation of the computational contribution of the BG to speech. Gradual pre- vs. post-DBS voice and speech changes are an important trend to highlight for patients’ quality of life (Tripoliti et al., 2011, 2014; Aviles-Olmos et al., 2014; Wertheimer et al., 2014; Tanaka et al., 2020; Gessani et al., 2023), but these gradual changes inform neurocomputational understanding of BG only indirectly because the neural substrates of such changes may result both from neuroplastic and neurodegenerative processes. We will thus focus primarily on short-term effects herein.

When considering the effects of STN-DBS on speech, a major confounding factor (from the perspective of understanding STN function) is that the STN is enveloped by axonal fibers, including motor efferents to the cranial nerves responsible for movements of the vocal tract and larynx residing within the internal capsule and ascending sensory fibers of the trigeminothalamic tract (Petersen et al., 2019). Given the thin morphology of the STN, it is difficult to avoid stimulating these fibers with DBS. GPi-DBS is less prone to this issue because GPi has a larger volume and therefore its borders are typically further from the internal capsule, and furthermore the trigeminothalamic tract is not nearby. The possibility of off-target stimulation highlights the need for caution when interpreting studies of STN-DBS that do not carefully control for electrode location (cf. Astrom et al., 2010; Jorge et al., 2020). In many cases speech impairment with STN-DBS may be due to disruption of signals in the internal capsule rather than (or in addition to) disruption of STN function. Furthermore, PD subtyping might elucidate why some patients experience a deterioration of speech after DBS while others experience an amelioration. Similarly, speech symptom subtyping may be a promising route to clarifying DBS’s effect on speech and making single-subject predictions about the risk of speech decline (Tsuboi et al., 2015; Tanaka et al., 2020; Gessani et al., 2023). A clear advantage of modern DBS is its programmability and dynamicity; closed-loop, responsive DBS systems will likely continue to improve DBS’s influence on speech (Little et al., 2016; Pina-Fuentes et al., 2020).

Setting these complications aside, the most pertinent studies for understanding the computational contribution of STN to speech are those that report effects on speech in a post-operative DBS-ON vs. DBS-OFF condition because this contrast reveals acute effects of DBS. One trend in these studies is that vocal parameters like loudness and pitch variability improve with DBS-ON (Dromey et al., 2000; Lundgren et al., 2011; Moreau et al., 2011; Karlsson et al., 2013; Skodda et al., 2014; Behroozmand et al., 2019) whereas speech intelligibility declines (Tornqvist et al., 2005; Klostermann et al., 2008; Tripoliti et al., 2008; Dromey and Bjarnason, 2011; Skodda et al., 2014). It is important to highlight the variability of patient outcomes in these studies; while the cohort statistics suggest one trend, many patients (~20% or more) will contradict the trend (Tripoliti et al., 2011; Tanaka et al., 2020; Gessani et al., 2023). Indeed, it is uncontroversial that STN-DBS increases the variability of speech outcomes (in both the short-term and long-term) relative to medication-only treatment.

Recently, Bobin et al. (2024) found that right STN stimulation was superior to left STN stimulation in improving dysphonia. They also report that with careful titration of stimulation parameters, both left and right STN stimulation could improve dysarthria acutely, with the effects of left STN stimulation limited to improvements in voice intensity measures. The authors also mapped stimulation location within the STN, finding posteromedial contacts had strong positive effects on voice parameters. This contrasts with a previous study reporting that *anterior* STN stimulation was associated with improved perceptual and acoustic-aerodynamic outcomes (Jorge et al., 2020), and another study reporting that posteromedial stimulation was associated with dysarthria (Astrom et al., 2010). Future studies reporting stimulation locations in greater quantity and with higher accuracy are needed to elucidate the extent to which STN topography and laterality contribute to voice and speech control.

Compared to STN-DBS, the influence of GPi-DBS on voice and speech has been less studied. A recent study comparing speech acoustic and perceptual measures from the speech of 10 STN-DBS and 8 GPi-DBS recipients found no differences between the two stimulation locations (van Brenk et al., 2024). A recent review article suggested that GPi-DBS may be favorable to STN-DBS along voice dimensions (Baudouin et al., 2023). Concordant with this idea, Finger et al. (2020) reported improved voice and speech intelligibility in patients undergoing bilateral GPi-DBS for dystonia. However, Chiu et al. (2020) looked retrospectively at 20 PD patients after bilateral GPi-DBS and found that laryngeal components along with velopharyngeal resonance worsened in the DBS-ON, medication-ON condition relative to the pre-operative medication-ON condition. While speech intelligibility remained unchanged in the short term (six-month follow-up vs. baseline), it trended downward after one year. These results are difficult to interpret, given the lack of a non-DBS comparison group to control for the expected effects of disease progression.

Thus far we have focused on the interaction between DBS and motor execution of speech. Another angle to consider is DBS and speech motor *learning*, motivated by the extensive evidence for the role of basal ganglia in songbird vocal learning (Haesler et al., 2007; Kosubek-Langer and Scharff, 2020), human and NHP non-speech motor learning (Doyon et al., 2009), and human speech acquisition (Lai et al., 2003; Vargha-Khadem et al., 2005; Ziegler and Ackermann, 2017). To our knowledge, no study has directly tested the effect of DBS-ON vs. DBS-OFF on speech motor learning in a laboratory setting, and results are mixed regarding whether PD patients with DBS realized greater gains from speech therapy than those without DBS (Spielman et al., 2011; Tripoliti et al., 2011). However, substantial evidence indicates a facilitative effect on short-term non-speech motor learning occurring during DBS-ON over DBS-OFF states. STN-DBS stimulation facilitates motor learning in tasks involving sequential cursor movements (Carbon and Eidelberg, 2006; Mure et al., 2012), sequential finger tapping (Muehlberg et al., 2023), single-target cursor movements (de Almeida Marcelino et al., 2019), and visuomotor perturbation adaptation (Singh et al., 2019). One study which measured regional cerebral blood flow during task performance found that STN-DBS-facilitated learning is associated with increased activity in lateral cerebellum and dorsal premotor cortex, coupled with reduced activity in supplementary motor area (Mure et al., 2012). This finding aligns with the prediction of the DIVA/GODIVA framework that successful motor sequence learning involves the chaining together of sequence elements via a cortico-cerebellar loop, along with reduced reliance on the SMA to control individual-element timing in well-learned sequences. GPi-DBS stimulation has been shown to improve both performance and learning of a sequential cursor movement task (Fukuda et al., 2002; Carbon and Eidelberg, 2006). Concurrent PET imaging revealed that GPi DBS-facilitated learning gains were associated with increased activity in a similar cortical network connected to STN DBS-facilitated learning, including bilateral premotor cortex (Fukuda et al., 2002). This network activity enhancement was not correlated with GPi-DBS facilitation of a motor reference task, indicating its specificity to motor learning. This learning-related network activity was subsequently shown to be associated with learning gains in the same task in healthy controls and PD patients without DBS treatment (Carbon et al., 2003). STN stimulation might additionally facilitate speech motor learning through an enhancement of verbal working memory (Pillon et al., 2000). The possibility of these effects holds clinical significance due to the widespread need of these patients for speech therapy to address disease-related speech and voice impairments (Ramig et al., 2008; Dressler et al., 2016; Barkmeier-Kraemer and Clark, 2017). It is noteworthy that DBS stimulation does not increase motor learning or performance to above the level of controls (Singh et al., 2019; Muehlberg et al., 2023), and the learning-facilitative effects of DBS may depend on disease progression being at an advanced stage (Meissner et al., 2016).

### 6.3 Pallidotomy

Stereotactic lesions to the basal ganglia have been used in various eras of neurosurgical treatment of PD (Guridi and Lozano, 1997). Popularized after a serendipitous surgical error, lesions to the globus pallidus (“pallidotomy”) have been shown to treat some of the most debilitating symptoms of PD (Cooper, 1959). Invasive vascular occlusion and chemical lesions (Guiot and Brion, 1953; Narabayashi et al., 1956; Obrador, 1957; Cooper, 1959), invasive thermal lesions (Svennilson et al., 1960; Laitinen et al., 1992), and most recently non-invasive focused ultrasound lesions (Krishna et al., 2023) share the same goal of destroying tissue in part of the pallidum. After the realization that dopamine-replacing therapies result in dyskinesias, thermal lesion unilateral and bilateral posteroventral pallidotomies (PVPs) were performed regularly in the 1990s and 2000s after Laitinen et al. (1992) reintroduced the procedure, integrating neuroimaging methods and anatomical knowledge (Laitinen et al., 1992; Iacono et al., 1995; Alterman and Kelly, 1998; Barlow et al., 1998; Scott et al., 1998; Ghika et al., 1999; Favre et al., 2000).

Given the many hypotheses of basal ganglia contributions to motor control (e.g., action selection, movement gain/vigor, habit formation, and behavioral automation), it is at first blush somewhat surprising that lesioning the primary output nucleus of the BG is not more detrimental to motor execution (Marsden and Obeso, 1994). As one of the most complex behaviors controlled by the human motor system, speech should, in theory, rely heavily on BG mechanism’s. However, speech production appears to remain largely intact in many patients even after bilateral PVP. In a rare study reporting acoustics and aerodynamics measurements before and after bilateral PVP in 14 patients, Barlow et al. (1998) found subtle changes along certain measures but overall “patients… were 100% intelligible before and after pallidotomy.” Favre et al. (2000) reported on both staged and simultaneous bilateral pallidotomies in a cohort of 17 patients. They found that 50–60% of patients experienced major speech deterioration after their procedure, though they speculate that speech problems involved the internal capsule rather than the GPi itself. De Bie et al. (2002) reported on 13 patients who underwent staged bilateral pallidotomy. Five of 13 patients “had problems with speech,” one of which had severe dysarthria. In a larger study with 115 patients in the UK and United states, Parkin et al. (2002) reported that “speech was adversely affected” after bilateral posteroventral pallidotomy but that “the change was small in most cases.” Two in three patients experienced a worsening of speech after pallidotomy, but only 8% of patients experienced major deterioration. Higuchi and Iacono (2003) looked retrospectively at 796 patients who underwent pallidotomy over a 7-year period. Of these, 272 received simultaneous bilateral pallidotomies while 88 received staged bilateral pallidotomies. They report that speech disturbances were observed in only 2.6% of patients after surgery, and that simultaneous bilateral pallidotomy was not significantly more associated with speech and swallowing disturbances than unilateral pallidotomy. However, some caveats are in order regarding older studies: the quality of imaging in these studies is generally lower that those performed with modern MRI sequences, the exact location and extent of the lesions are less precisely reported, and the durability of the behavioral effect (an indirect measure of lesion permanence) may be unknown. In a more recent study, York et al. (2007) reported speech declines in 9 of 15 patients who underwent bilateral staged pallidotomy at 3–12 months after their second procedure, with speech reported as the most prevalent complication at long-term follow-up as 79% of patients experienced hypophonia at >2 years post-procedure.

Pallidotomy fell out of favor with the rise of DBS in the late 2000s, but it has seen a resurgence with novel non-invasive technology. Focused ultrasound ablation of the globus pallidus is now in clinical trial for PD patients averse to invasive, open-brain surgery. Krishna et al. (2023) conducted a double-blind clinical trial of 65 PD patients who underwent unilateral focused ultrasound pallidal ablation, and 22 patients assigned to a sham procedure. Although just 2 of 65 patients experienced noticeable dysarthria after the procedure, no other speech measures were assessed.

Although rates of speech complications vary greatly across surgical centers after PVP (Favre et al., 2000), the relatively mild impact on speech from bilateral pallidotomy in some patients appears to indicate that the role(s) of the basal ganglia in speech may be supportive rather than essential, though one cannot completely rule out the possibility that spared portions of the BG output nuclei (e.g., the non-lesioned portion of GPi) or the substantia nigra pars reticulata (SNr) carry out speech-related roles after bilateral PVP. One possible explanation is that the BG are heavily involved in the learning of motor sequences, but their role diminishes after sequence learning, at which point they may still play a role in movement gain but are no longer needed for sequencing or action selection (Turner and Desmurget, 2010).

## 7 Summary

In this article we reviewed the scientific literature as it concerns hypothesized roles of the basal ganglia in speech production and how these roles may be impacted by Parkinson’s disease. The possible roles, summarized in the following paragraphs, include modulating the gain of speech movements, contributing to the learning of frequently used speech movement sequences, and contributing to the fluent sequencing of words within longer utterances.

Regarding gain modulation, widespread reports of reduced movement amplitudes and vocal output in PD, along with reduced movement gains found with pallidotomy, indicate a BG role in movement gain. Within the DIVA/GODIVA framework, this reduced gain is attributed to reduced support from the cortico-BG loop for the current word’s node in an SMA-based initiation map, which in turn reduces the gain of the motor commands constituting the word’s motor program. According to this account, this reduced support can also result in early termination of the word’s motor program, thus providing an explanation for common reports of accelerating speech in PD patients, a finding that might otherwise seem at odds with reduced movement gains.

A BG role in the learning of motor sequences has been firmly established in animal studies of non-speech motor sequencing. Because speech motor sequence learning (in the form of word learning) primarily occurs well before the onset of PD, an impairment in this capacity may go unnoticed unless the patient is learning a second language. The DIVA/GODIVA framework predicts that PD patients will show significant deficits relative to age-matched controls in learning to produce new words or word sequences; this prediction is supported by several experimental findings concerning the learning of novel speech sequences by individuals with PD.

A third possible role of the BG in speech concerns the temporary buffering and sequential readout of phonological material for longer utterances such as sentences. Speech sequence-related activity has been observed in STN during production of novel syllable sequences in a task that did not involve motor learning, suggesting some role for the BG in sequencing of words within longer utterances. However, bilateral pallidotomy does not typically result in a significant increase in sequencing errors, suggesting that any such role is supplementary rather than central. Numerous studies have reported reduced word stress in PD; these findings are compatible with the view that the BG are heavily involved in controlling movement intensity based in part on motivational context.

Although the primary roles of the BG within the DIVA/GODIVA framework concern the feedforward control mechanisms described above, a number of studies have investigated sensory-motor processes, specifically auditory and somatosensory feedback control, in PD. A number of studies report little or no impairment in pure auditory processing (with the exception of loudness and duration, two aspects of speech that are also impaired in the motor output of speakers with PD) nor in auditory feedback control of speech as measured by reflexive responses to unpredictable auditory perturbations which invoke auditory feedback control mechanisms, though reduced responses to F1 perturbations in the medication OFF state have been reported. Instead, PD patients often show exaggerated reflexive responses, possibly due to a reduced gain in the feedforward control subsystem that would otherwise dampen the auditory feedback control response. These findings are consistent with the DIVA/GODIVA framework, which posits that the BG are not heavily involved in sensory feedback-based control of speech movements. The frequently reported finding of reduced audiomotor adaptation in PD indicates a role for dopamine and/or cortico-BG loops in stimulating learning of feedforward commands; this role is not yet explicitly included in the DIVA/GODIVA framework.

Studies utilizing somatosensory perturbations, such as speaking with a bite block, also report exaggerated responses in PD. Unlike auditory feedback, which does not depend heavily on motor commands, somatosensory feedback processing requires integration of (possibly impaired) motor information with tactile and proprioceptive information from mechanoreceptors in the vocal tract articulators. One possible explanation for increased reflexive responses to tactile stimulation in PD is that the somatosensory feedback control system acts with a higher-than-normal gain. However, this account cannot explain experimental data indicating that PD speakers actually increase their vowel contrasts (compared to unperturbed speech) when speaking with a bite block; in other words, they overshoot their normal vowel targets. This finding may indicate that PD speakers underestimate the size of their corrective movements under perturbed conditions, providing an alternative account of the increased reflexive responses in response to auditory and somatosensory perturbations in PD.

The literature on therapeutic effects of drug and surgical treatments provides further clues to BG involvement in speech. Dopaminergic replacement therapy does not appear to consistently affect speech, though a few studies show improvement in some acoustic measures of speech. The literature on deep brain stimulation is even more mixed, with some studies showing improvement (particularly in loudness and pitch variability), but many studies noting deterioration of speech with DBS. In many cases deterioration of speech with DBS likely arises due to unintended stimulation of descending motor tracts in the internal capsule, or in the case of the STN, ascending sensory tracts in the medial lemniscus. In some cases, deterioration of speech occurs immediately, while in others it occurs only over a period of months where the effects of disease progression are difficult to disentangle. The literature on pallidotomy suggests relatively little deterioration of speech even with bilateral pallidotomy; this finding strongly suggests that any role played by the BG in executing previously learned movements is supplementary rather than mandatory, perhaps limited to adjustment of movement gains and durations based (at least in part) on motivational factors.

## Data availability statement

The original contributions presented in the study are included in the article/supplementary material, further inquiries can be directed to the corresponding author.

## Author contributions

JM: Writing – original draft, Writing – review & editing. LB: Writing – original draft, Writing – review & editing. AM: Writing – original draft, Writing – review & editing. RT: Writing – original draft, Writing – review & editing. RR: Writing – original draft, Writing – review & editing. FG: Writing – original draft, Writing – review & editing.

## Footnotes

^1^For the remainder of this article, we will assume word-sized chunks and use the term “words” for the sake of readability, with the understanding that other chunk sizes (e.g., syllables, phonological words) may be represented in addition to, or instead of, word-sized chunks.

^2^Hereafter, hemispheric laterality will only be mentioned in cases where function is significantly lateralized.

^3^Although the cortical areas depicted in Figure 4 all project to the striatum, they do not all converge on the same portion of the striatum. For example, the putamen targets of prefrontal areas such as preSMA and pIFS are located dorsomedially relative to the more “motoric” regions vMC, SMA, and vPMC (Nambu, 2011). The theoretical account described herein is not dependent on any topographic organization within BG structures; however, it is certainly possible that such an organization may confer performance advantages over non-topographic (e.g., randomly distributed target neurons in the striatum) representations, and disruption of this topography may contribute to motor symptoms in PD (Nambu, 2011).

^4^Not shown in Figure 4 are limbic and paralimbic areas that also contribute to what can be termed the *motivational context* (*cf.* Mazzoni et al., 2007); these will not be addressed in detail here since these aspects of the circuit are not elaborated in the DIVA or GODIVA model at present.

^5^Turner and Desmurget (2010) use the term vigor to indicate an interaction between a motivational signal and movement gain. Since the DIVA/GODIVA framework does not address the motivational component, we will use the word gain in the remainder of the article in place of the term vigor.

^6^It is important to consider that the variability of speech rate presentations in PD may reflect different neural mechanisms. For example, slow speaking rate can be the result of cognitive decline rather than purely motor processes (Lowit et al., 2006). Variability in speech rate symptoms may also reflect broader impairments in temporal processing (Jones and Jahanshahi, 2014), internal monitoring of speech rate (Ackermann et al., 1997), or an impaired ability to control the duration of speech events (Ludlow et al., 1987).

^7^The other common target of DBS for movement disorders is ventral intermediate nucleus (Vim) of the thalamus (Neudorfer et al., 2024). Vim is targeted for treatment of essential tremor. Vim receives projections primarily from the cerebellum rather than the basal ganglia (Ilinsky et al., 2018); it will therefore not be treated here.
